# Clinical, Pathological, and Molecular Features of Lung Adenocarcinomas with AXL Expression

**DOI:** 10.1371/journal.pone.0154186

**Published:** 2016-04-21

**Authors:** Katsuaki Sato, Kenichi Suda, Shigeki Shimizu, Kazuko Sakai, Hiroshi Mizuuchi, Kenji Tomizawa, Toshiki Takemoto, Kazuto Nishio, Tetsuya Mitsudomi

**Affiliations:** 1 Division of Thoracic Surgery, Department of Surgery, Kinki University Faculty of Medicine, Osaka-Sayama, Japan; 2 Department of Pathology, Hyogo College of Medicine, Hyogo, Japan; 3 Department of Genome Biology, Kinki University Faculty of Medicine, Osaka-Sayama, Japan; Univesity of Texas Southwestern Medical Center at Dallas, UNITED STATES

## Abstract

The receptor tyrosine kinase AXL is a member of the Tyro3-Axl-Mer receptor tyrosine kinase subfamily. AXL affects several cellular functions, including growth and migration. AXL aberration is reportedly a marker for poor prognosis and treatment resistance in various cancers. In this study, we analyzed clinical, pathological, and molecular features of AXL expression in lung adenocarcinomas (LADs). We examined 161 LAD specimens from patients who underwent pulmonary resections. When AXL protein expression was quantified (0, 1+, 2+, 3+) according to immunohistochemical staining intensity, results were 0: 35%; 1+: 20%; 2+: 37%; and 3+: 7% for the 161 samples. AXL expression status did not correlate with clinical features, including smoking status and pathological stage. However, patients whose specimens showed strong AXL expression (3+) had markedly poorer prognoses than other groups (*P* = 0.0033). Strong AXL expression was also significantly associated with downregulation of E-cadherin (*P* = 0.025) and CD44 (*P* = 0.0010). In addition, 9 of 12 specimens with strong AXL expression had driver gene mutations (6 with *EGFR*, 2 with *KRAS*, 1 with *ALK*). In conclusion, we found that strong AXL expression in surgically resected LADs was a predictor of poor prognosis. LADs with strong AXL expression were characterized by mesenchymal status, higher expression of stem-cell-like markers, and frequent driver gene mutations.

## Introduction

Lung cancer is the leading cause of cancer-related mortality in developed countries [1], and lung adenocarcinoma (LAD) is the most common histologic subtype in lung cancer [2]. In some LADs, activation of receptor tyrosine kinases has a key function in carcinogenesis and maintenance of cancer phenotypes. For example, some LADs are driven by somatic mutations in the epidermal growth factor receptor (*EGFR*) gene; these tumors are highly responsive to EGFR tyrosine kinase inhibitors (TKIs). On the other hand, amplification of the *MET* gene or *ERBB2* gene reportedly lead to acquired resistance to EGFR-TKIs in LADs with *EGFR* mutation [3, 4]. Further understanding of LADs therefore requires analysis of the roles of other receptor tyrosine kinases in addition to those with driver mutations.

AXL receptor tyrosine kinase, which is also known as ARK, JTK11, or Tyro7, is a member of the Tyro3-Axl-Mer receptor tyrosine kinase subfamily. AXL transduces signals from the extracellular matrix into the cytoplasm by binding to the vitamin K-dependent protein growth arrest-specific-6 (Gas6) [5]. AXL is involved in several cellular functions including growth, migration, aggregation and anti-inflammation, which are also associated with cancers. Recent studies AXL to be a marker for poor prognosis in lung cancers [6], breast cancers [7], pancreatic cancer [8], renal cell carcinoma [9] and ovarian cancer [10], and for treatment resistance in lung cancers [11–13], gastrointestinal stromal tumor [14], esophageal cancer [15], and ovarian cancer [16]. Although AXL’s association with poor prognosis in LAD has been reported [6], the clinical, pathological, and molecular characteristics of AXL^+^ LAD remains unclear.

Here, we explored clinical, pathological and molecular characteristics of AXL^+^ LAD.

## Materials and Methods

### Patient cohort

Between January 2007 through April 2009, a total of 169 patients underwent pulmonary resection for primary LAD at the Division of Thoracic Surgery, Department of Surgery, Kinki University Faculty of Medicine. Tissue samples were obtained from patients who had tumors of 1 cm or larger in diameter (*n* = 161). All of these tumors were histologically confirmed as LADs, and their lymph node metastatic status, pathological staging, and degree of differentiation were assessed. Staging by the latest tumor-node-metastasis classification (UICC ver.7) was used [17]. Since many of the patients were already dead or lost to follow-up, we posted information on this research plan on our website (http://www.kindai-geka.jp/biomarker/2013/07/post-2.html) for those from whom informed consent could not be obtained. We also provided an opportunity of exclusion of their samples from the analyses upon their request through the website, according to the instruction of the IRB. This study was reviewed and approved by the Ethics Committee of the Faculty of Medicine at Kinki University.

### Pathologic evaluation

Predominant histologic patterns were determined by two of us (KS, SS), according to the new WHO classification system [17]. Grade classification was also performed based on the new WHO classification [17]. High grade included micropapillary and solid predominant tumors and invasive mucinous adenocarcinomas; low grade included lepidic, papillary, and acinar predominant adenocarcinomas [18, 19].

### Immunohistochemistry (IHC)

Tissue microarrays (TMA) were created by aligning 2-mm cores (2 cores from each specimen including the part with the predominant histologic pattern) taken from paraffin-embedded tumor blocks. IHC staining was performed using the TMA. Briefly, each TMA was cut into 4-μm sections and mounted on glass slides. After deparaffinization and rehydration, the slides were heated in a retrieval solution (Dako Real Target Retrieval Solution, Dako, Tokyo, Japan) for antigen retrieval at 121°C for 15 min. After quenching endogenous activity with 3% hydrogen peroxide for 30 min, the sections were treated with blocking agent (Dako Protein Block. Serum-Free. Ready-To-Use, Dako, Tokyo, Japan) for 10 min to eliminate nonspecific staining. The sections were incubated overnight with an anti-AXL antibody (AF154, 1:100, R&D Systems Inc., MN, USA). The slides were then incubated for 60 min with the secondary antibody (N-Histofine Simple Stain™ Max PO (G), Nichirei, Tokyo, Japan), followed by visualization with 3,3′-diaminobenzine tetrahydrochloride (Dako Liquid DAB + Substrate Chromogen System, Dako, Tokyo, Japan). Finally, the sections were counterstained with hematoxylin.

Other primary antibodies; E-cadherin (#3195, 1:500, Cell Signaling Technology, Danvers, MA, USA), vimentin (#5741, 1:200, Cell Signaling Technology), CD44 (#3570S, 1:500, Cell Signaling Technology), ALDH1A1 (ab52492, 1:500, Abcam, Cambridge, MA, USA), and P-glycoprotein (ab3366, 1:100, Abcam) were also used to evaluate expression of these proteins. Dako Real Envision Detection Reagent Peroxidase Rabbit/Mouse was used as secondary antibody.

Expression of AXL and other proteins was scored by staining intensities, graded as 0 (no staining), 1+ (weak), 2+ (moderate), or 3+ (strong). For AXL expression, we used vascular endothelial cells as an internal control. Staining intensity similar to that of vascular endothelial cells was classified as 3+, and weaker than that of vascular endothelial cells was classified into 1+ or 2+. Weak partial membrane staining was defined as 1+, and weak to moderate complete membrane staining was defined as 2+, according to Ishikawa et al[6]. We independently analyzed two cores for each tumor specimen. When scores of the two cores were different, we adopted the higher one. Scoring was performed by two of us (KS and SS) who were blinded to patients’ clinical information.

Expression status was classified as positive if more than 10% of cells showed staining (vimentin, CD44, ALDH1A1 and P-glycoprotein) and E-cadherin downregulation was defined as negative staining in more than 10% of cells, following previous reports [20, 21].

### Target sequencing analysis

Target sequencing analysis was performed as previously described [22]. Briefly, 10 ng of genomic DNA was extracted from formalin-fixed paraffin-embedded sections, and used for multiplex PCR amplification with Ion AmpliSeq Library kit 2.0 (Life Technologies) and the Ion AmpliSeq Cancer Hotspot panel v2 (Life Technologies). The Ion Xpress Barcode Adapters (Life Technologies) were ligated into the PCR products and purified with Agencourt AMPure XP beads (Beckman Coulter, Brea, CA). The purified libraries were then pooled and sequenced on an Ion Torrent PGM device (Life Technologies) using the Ion PGM 200 Sequencing kit v2 (Life Technologies) and the Ion 318 v2 Chip kit.

DNA sequencing data were accessed through the Torrent Suite v4.0 software program. Reads were aligned against the hg19 human reference genome; variants were called using the variant caller v4.0. Raw variant calls were filtered out using the following annotations: homozygous and heterozygous variants, quality score of <100, depth of coverage <19. Germline mutations were excluded using the Human Genetic Variation Database (http://www.genome.med.kyoto-u.ac.jp/SnpDB) [23].

### Statistical analysis

Statistically significant differences within categorical data were evaluated by the χ^2^ test or Fisher exact test. The Cochran–Armitage test was used for categories with trends. Overall survival was defined as the time from pulmonary resection to death. Patients without a known date of death were censored at the time of the last follow-up. Kaplan–Meier curves were used to estimate survival probability at each time point; the log-rank test was used to compare differences between groups. Univariate and multivariate analysis of overall survival used the Cox proportional hazard modeling technique. All statistical analyses were performed with JMP version 11 (SAS Institute). *P* < 0.05 was considered significant.

## Results

### AXL expression and clinical factors

**Fig 1:**
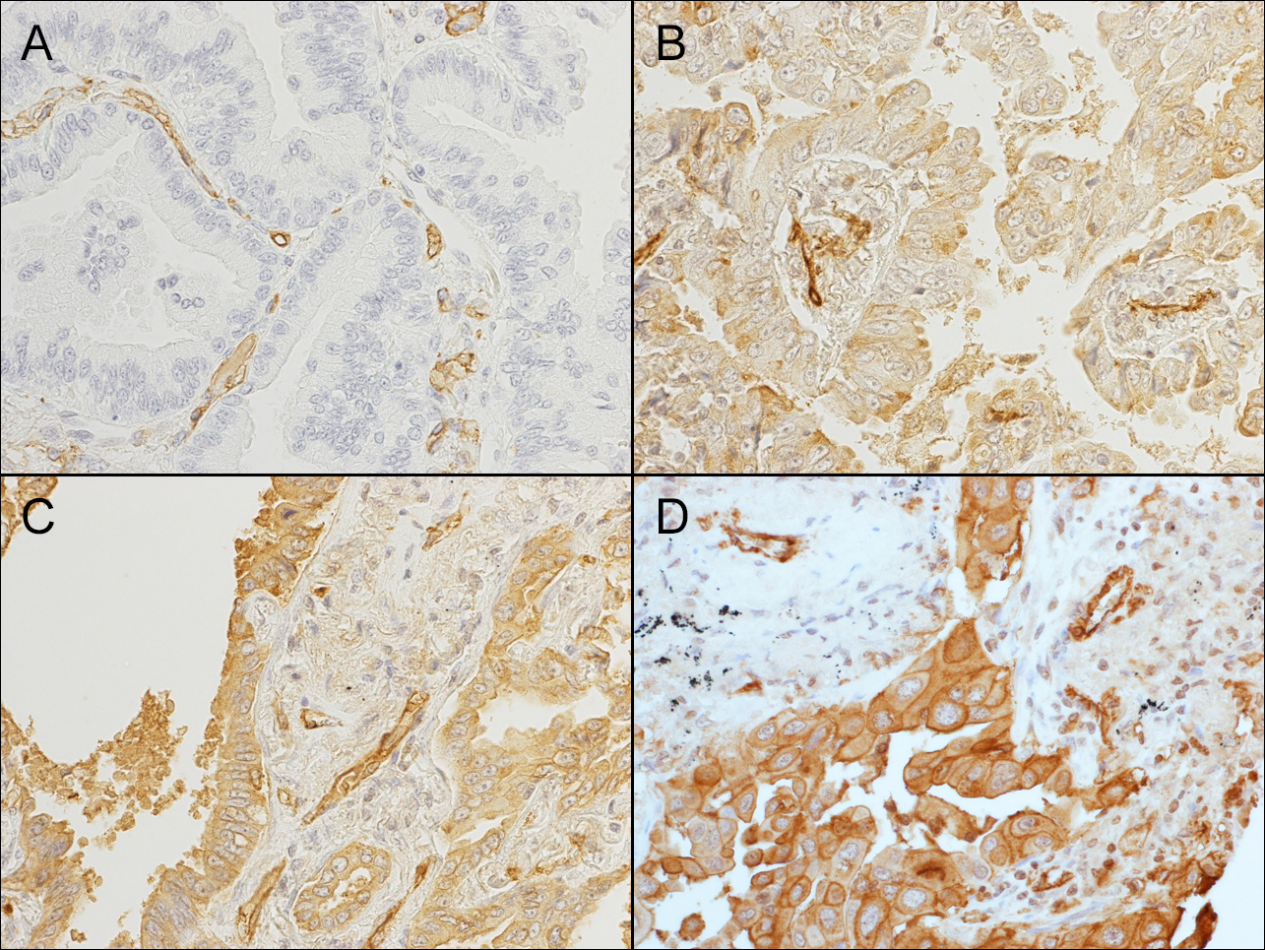
AXL expression in LADs by IHC. AXL expression status was classified in four categories, 0 (negative, A), 1+ (weak, B), 2+ (moderate, C), and 3+ (strong, D). All images include vascular endothelial cells, which were used as internal controls.

Representative AXL staining patterns are shown in Fig 1. Of the 161 specimens, 57 had AXL expression level 0 (Fig 1A), 32 had 1+ (Fig 1B), 60 had 2+ (Fig 1C), and 12 had 3+ (Fig 1D). None of the clinical factors (sex, age, carcinoembryonic antigen, stage, smoking status, pathologic grade) were significantly associated with AXL expression (Table 1). There was heterogeneity in AXL expression in our cohort. However the scores of two cores in each tumor were similar; 91.7% of tumors showed the same or 1 point difference between the two cores.

**Table 1:** Relationships between AXL expression and clinical factors in 161 LAD specimens.

Factor			AXL expression				P*
			0	1+	2+	3+	
		*n*	*n* = 57	*n* = 32	*n* = 60	*n* = 12	
Sex	Female	87	31 (35%)	17 (20%)	33 (38%)	6 (7%)	0.77
	Male	74	26 (35%)	15 (20%)	27 (37%)	6 (8%)	
Age (years)	< 69	82	26 (32%)	20 (24%)	30 (37%)	6 (7%)	0.95
	≥ 69	79	31 (39%)	12 (15%)	30 (38%)	6 (8%)	
CEA	< 5.0	103	38 (37%)	18 (17%)	41 (40%)	6 (6%)	0.25**
	≥ 5.0	54	17 (31%)	14 (26%)	17 (31%)	6 (12%)	
	unknown	4	2 (50%)	0 (0%)	2 (50%)	0 (0%)	
P-stage	I	111	40 (36%)	24 (22%)	38 (34%)	9 (8%)	0.82
	II	25	9 (36%)	3 (12%)	12 (48%)	1 (4%)	
	III	23	8 (35%)	5 (22%)	8 (35%)	2 (8%)	
	IV	2	0 (0%)	0 (0%)	2 (100%)	0 (0%)	
Smoking	Ever	78	27 (35%)	20 (26%)	28 (36%)	3 (3%)	0.084**
	Never	72	27 (38%)	11 (15%)	26 (36%)	8 (11%)	
	Unknown	11	3 (27%)	1 (9%)	6 (55%)	1 (9%)	
Grade***	Low	129	44 (34%)	28 (22%)	48 (37%)	9 (7%)	0.62
	High	31	12 (39%)	4 (13%)	12 (38%)	3 (10%)	

*[0/1+/2+] vs [3+].

**Patients with unknown status were excluded from statistical analysis.

***One case of minimally invasive adenocarcinoma was excluded. CEA: carcinoembryonic antigen, in μg/L.

### AXL expression and prognosis

**Fig 2:**
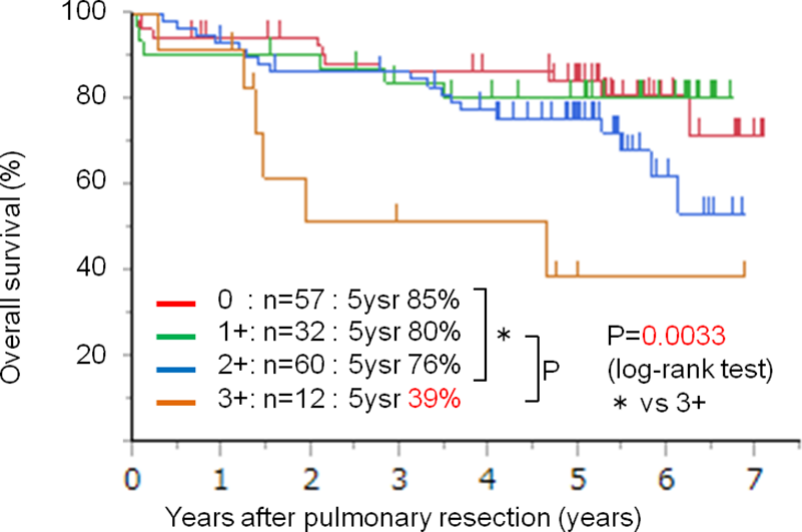
Overall survival of the patients based on AXL expression status. Overall survival rates correlated with AXL expression status. The group of patients whose specimens showed strong AXL expression (3+ intensity) showed markedly shorter survival than groups whose specimens showed weaker intensity (0, 1+ or 2+; *P* = 0.0033; log-rank test). 5ysr: 5-year survival rates.

We then compared overall survival among groups with different AXL expression status. Five-year survival rates by AXL expression scores were 0: 85%; 1+: 80%; 2+: 76%; and 3+: 39% (Fig 2); in particular, 5-year survival differed very significantly for patients in the AXL 3+ group compared with other groups (*P* = 0.0165). In multivariate analyses of age, sex, smoking status, and pathological stage, AXL 3+ expression was also a significant predictor of poor prognosis (*P* = 0.048, data not shown). We focused on the 3+ group in because of poor prognostic implications of 3+ AXL.

### AXL strong expression and clinical / molecular factors

We compared clinical characteristics of patients with AXL 3+ LAD specimens with the other groups, but found 3+ AXL expression was not significantly correlated with any of the analyzed clinical factors, including sex, smoking status, and P-stage.

We then analyzed correlations between AXL 3+ expression and several molecular markers that reportedly confer poor prognosis in lung cancers. In analyses for epithelial–mesenchymal transition (EMT) markers and so-called cancer stem cell (CSC) markers, we observed significant correlation between AXL 3+ expression and these molecular markers. Of the 12 patients with AXL 3+ specimens, 7 (58%) showed down-regulated E-cadherin, compared with only 26% of the 0/1+/2+ group (*P* = 0.025; Table 2). In addition, AXL 3+ expression tended to be correlated with vimentin expression, although not significantly so (3+: 67% vs 0/1+/2+: 43%; *P* = 0.11). On the other hand, the CD44^+^ rate was significantly higher in the AXL 3+ group (83%) than in the 0/1+/2+ counterpart (36%; *P* = 0.0010), and p-glycoprotein expression was relatively higher in the AXL 3+ group (*P* = 0.069).

**Table 2:** Relationships between AXL expression and markers for EMT and CSCs.

Factor		AXL expression		*P*
		0/1+/2+	3+	
		*n* = 149	*n* = 12	
E-cadherin	Normal	110 (74%)	5 (42%)	0.025*
	Downregulated	39 (26%)	7 (58%)	
Vimentin	Negative	85 (57%)	4 (33%)	0.11
	Positive	64 (43%)	8 (67%)	
CD44	Negative	96 (64%)	2 (17%)	0.0010*
	Positive	53 (36%)	10 (83%)	
ALDH1A1	Negative	59 (40%)	5 (42%)	0.89
	Positive	90 (60%)	7 (58%)	
P-glycoprotein	Negative	77 (52%)	3 (25%)	0.069
	Positive	72 (48%)	9 (75%)	

*Significant relationship

### AXL strong expression and genetic aberrations

We searched for mutations in 22 cancer-related genes, using target sequencing techniques to further characterize the 12 patients who had AXL 3+ expression, of whom 6 had mutations in *EGFR*, 2 in *KRAS*, and 1 *EML4–ALK* fusion gene (Table 3). Among the other patients, 2 harbored *TP53* mutations and 1 harbored a *SMAD4* mutation. These results indicate that most AXL 3+ tumors have so-called “driver mutations.”

**Table 3:** Results of target sequencing for 22 genes in AXL 3+-expressing lung adenocarcinomas from 12 patients.

Case	P-stage	Mutation of interest	Rec	TKI	Cx
1	IB	*EGFR* (19del)	−	−	−
2	IIIA	*EGFR* (L858R)	+	Gefitinib	−
3	IB	*EML4-ALK*	+	Gefitinib	+
4	IB	*EGFR* (exon 20INS)	+	−	−
5	IIIA	*KRAS* (G12D)	−	−	−
6	IA	*EGFR* (L858R, T790M)	−	−	−
7	IB	*KRAS* (G12C)	−	−	−
8	IB	*EGFR*(19del)	+	−	−
9	IIA	*TP53*	+	−	+
10	IB	*EGFR* (L858R)	+	−	+
11	IB	*SMAD4*	+	−	−
12	IA	*TP53*, *PIK3CA*, *DDR2*	+	−	−

Cx: chemotherapy; Rec: recurrence; TKI, tyrosine kinase inhibitor

### A case of LAD with AXL strong expression who treated by gefitinib

**Fig 3:**
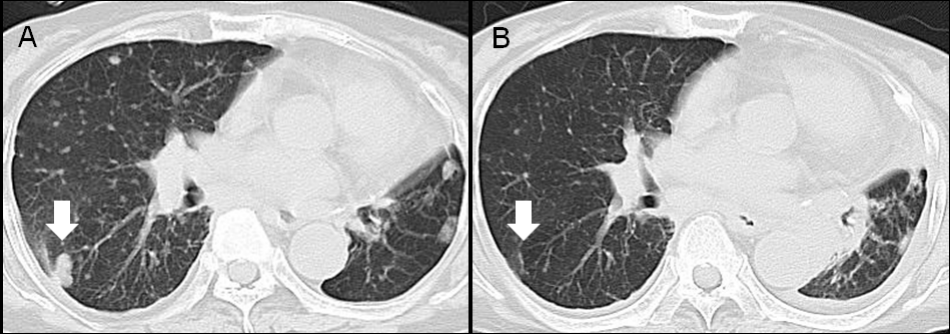
A case of AXL 3+-expressing lung adenocarcinoma that responded well to gefitinib. (A) Pre-treatment and (B) post-treatment (6 months after initiation of gefitinib, CT scans are shown.

Among 7 patients whose AXL 3+-expressing adenocarcinomas had “targetable” mutations, only one patient with an *EGFR* mutation received an appropriate molecular targeted drug for her recurrent disease (Case 2). Although AXL expression reportedly confers acquired resistance to *EGFR* tyrosine kinase inhibitors, this patient responded well to gefitinib, as shown in Fig 3.

## Discussion

In this study, we found that patients’ prognoses were inversely correlated with AXL expression, as previously shown by Ishikawa, et al. [6]. However, we observed no correlation between AXL expression status and clinical factors such as sex, smoking history, or pathological stage. This was inconsistent with the analysis of Ishikawa et al., which showed correlations between AXL expression and younger age (*P* = 0.022), female sex (*P* = 0.018), and advanced P-stage (*P* < 0.001) [6]. Our molecular analysis found that AXL 3+-expression was correlated with the presence of so-called “driver mutations” such those found in *EGFR*, *KRAS* and *ALK* fusion genes. Because these molecular aberrations reportedly correlate with female sex, younger age, and advanced disease, respectively [24], these discrepancies may reflect the complexity of clinical and molecular characteristics of AXL 3+ LADs.

In LAD, changes resulting from driver mutations generally occur in a mutually enforcing fashion [25]. Our finding that AXL 3+ expression often co-exists with driver gene mutations suggests that AXL may play supplementary or modifying roles in these changes. The frequency of these driver mutations were identical to the previous report that analyzed unselected Japanese lung adenocarcinoma patients; EGFR mutation, KRAS mutation, ALK fusion, or HER2 mutation were identified in 67.7% (216/319) [26].

The IHC analyses showed that AXL expression in LADs was correlated with EMT status, which was consistent with the previous reports [12, 27]. In addition, we found a correlation between strong AXL expression and that of CSC-related proteins. This is a novel finding in lung cancer, as far as we know. Although AXL has been shown to mediate stem-like behavior in other solid tumors such as breast cancer, glioblastoma, and cutaneous squamous cell carcinoma [28–30], this is a new finding in lung cancer. Because tumors with stem-like features have been shown to lead to poor survival and resistance to therapies, AXL strong expression in our lung cancer patients may promote such features and contribute to their poorer prognosis.

AXL activation has been recently reported to confer acquired resistance to EGFR-TKIs in lung cancers with *EGFR* mutations [12, 31, 32]. However a patient in this study, whose LAD specimen had 3+ AXL expression and an *EGFR* mutation, responded well to gefitinib. Because she died of another disease, progression-free survival was unclear in her case. Our analysis implies that strong AXL expression does not preclude EGFR-TKI treatment.

A limitation of our analysis is the effect of tumor heterogeneity, because we utilized TMA tissue specimens. To check the heterogeneous status of AXL expression, we independently analyzed two cores for each tumor specimen, and adopted the higher score, as described in Methods. However, as described in Results, heterogeneity was negligible.

In conclusion, strong AXL expression in resected LAD specimens is marker for poor prognosis. Lung adenocarcinomas with strong AXL expression may be characterized by mesenchymal status, higher expression of CSC markers, and frequent presence of driver gene mutations.
